# Reply to Comment on “The effectiveness of home versus community-based weight control programmes initiated soon after breast cancer diagnosis: a randomised controlled trial”

**DOI:** 10.1038/s41416-019-0715-z

**Published:** 2020-01-14

**Authors:** Michelle Harvie, Mary Pegington, Nigel Bundred, Anna Campbell, John Belcher, Sacha Howell, Anthony Howell

**Affiliations:** 1 Prevent Breast Cancer Research Unit, The Nightingale Centre, Manchester University NHS Foundation Trust, Manchester, UK; 2 Manchester Breast Centre, Manchester Cancer Research Centre, University of Manchester, Manchester, UK; 3 Division of Cancer Sciences, Faculty of Biology, Medicine and Health, University of Manchester, Manchester, M13 9PL UK; 4 School of Applied Sciences, Edinburgh Napier University, Edinburgh, UK; 5 Department of Medical Statistics, Manchester University NHS Foundation Trust, Manchester, UK; 6 The Christie NHS Foundation Trust, Manchester, UK

**Keywords:** Weight management

## Mixed BMI groups

Our programme aimed to prevent weight gain in healthy weight women and reduce weight in women who were overweight/obese since both may improve outcome and well-being after a diagnosis of breast cancer (BC).^1,2^ Women in the community arm received initial one-to-one dietetic advice, before entering mixed body mass index (BMI) community groups. Healthy weight women were advised to meet estimated energy requirements, while overweight/obese women were advised to follow a 25% energy restriction to achieve weight loss.

We acknowledge the concern that heavier women may not feel comfortable in mixed BMI groups, as previously reported in weight loss focussed programmes.^3^ However, our programme was framed as a weight loss/weight gain prevention lifestyle programme to improve future health and quality of life after a diagnosis of BC. There was no evidence of disengagement among heavier women in our study. Mean attendance for group sessions among healthy weight, overweight and obese participants was 64%, 66% and 62%, respectively. In-depth interviews cited advantages in meeting others who were experiencing similar cancer-related problems. Thus, highlighting the importance of the BC diagnosis rather than their weight to the salience of their current situation.

Lifestyle behaviour change programmes in other health settings commonly include subjects of mixed BMI, for example, the UK Diabetes Prevention Programmes includes individuals with haemoglobin A1c > 42 mmol/mol, of whom 27% are a healthy weight, 39% overweight and 33% obese.^4^ Similarly, cardiac rehabilitation programmes include individuals who are a healthy weight (24%), overweight (37.3%) and obese (38%).^5^

## Analyses

We did not use multiple imputation since this usually assumes data is missing at random (MAR). MAR was unlikely in the B-AHEAD study, as non-attendance at study appointments was more likely for those who had gained weight. We accept that single imputation approaches are limited and advised generally against for missing data. Analysis of longitudinal studies should consider missing data at each stage of the research and include appropriate sensitivity analysis of the missing data mechanism as highlighted recently by Bell et al.^6^

## Timepoints

The combined weight change outcome was pragmatic to provide an overall estimate of the programme for all early BC patients. We have also presented changes for the healthy and overweight/obese sub-groups. The 6-month assessment was timed to occur after the end of adjuvant treatments to maximise attendance. It also offers a better reflection of sustained behaviour change beyond the intensive intervention phase.

## Intervention timing

Our study tackled weight control soon after diagnosis and during adjuvant treatment. There is debate, but few data on the best time to initiate lifestyle behaviour change.

Optimum timing is based on a number of key criteria. First, when are weight loss/dietary interventions most clinically or biologically relevant? When are patients most likely to engage with programmes? Are there times when patients have difficulties to change behaviours because of the challenges of treatment or other issues? Is intervention timing key to long-term maintained behaviours?

Our intervention at diagnosis aimed to offset deleterious gains in fat and loss of lean body mass, which can occur during adjuvant treatments. Bail and colleagues^7,8^ question whether our uptake could have been greater if we had approached patients after treatment, citing higher uptakes for weight loss studies, which recruited post treatment. Comparing uptakes across studies is difficult, since this depends on the initial mode of patient identification. The LISA trial reported that 80% of women identified in their oncology centres consented to trial screening.^7^ However, it is not clear whether they approached all eligible women, or used a selective approach thus yielding superior recruitment. The Energy Study mailed 14,051 women to recruit 697 women, hence a 5% uptake.^8^ No studies have compared uptake at diagnosis vs. post treatment. The individual nature of cancer patients necessitates a range of timings and approaches to maximise engagement with lifestyle behaviour change.

Physical activity interventions appear to be effective both during and after active cancer treatments. While dietary interventions have a lower success during active treatment,^9^ which is consistent with the poorer weight control success among B-AHEAD participants receiving adjuvant chemotherapy.

The B-AHEAD study does not indicate that time of diagnosis is the optimal teachable moment for patients with early BC. However, it highlights the feasibility of engaging significant numbers of patients at this often overlooked/avoided time point, regardless of BMI or adjuvant therapies.

Our programme was successful for preventing weight gain among healthy weight women and for achieving weight loss among those with overweight/obesity not receiving adjuvant chemotherapy. Weight control and energy restriction may be specifically important among patients receiving adjuvant chemotherapy. Our B-AHEAD 2 and other ongoing studies are testing whether more achievable approaches such as intermittent energy restriction are effective among these patients.^10^

## Data Availability

Not applicable.
